# Toward a Stochastic Complete Active Space Second-Order Perturbation Theory

**DOI:** 10.1021/acs.jpca.3c05109

**Published:** 2023-12-28

**Authors:** Arta A. Safari, Robert J. Anderson, Giovanni Li Manni

**Affiliations:** Max-Planck-Institute for Solid State Research, 70569 Stuttgart, Germany

## Abstract

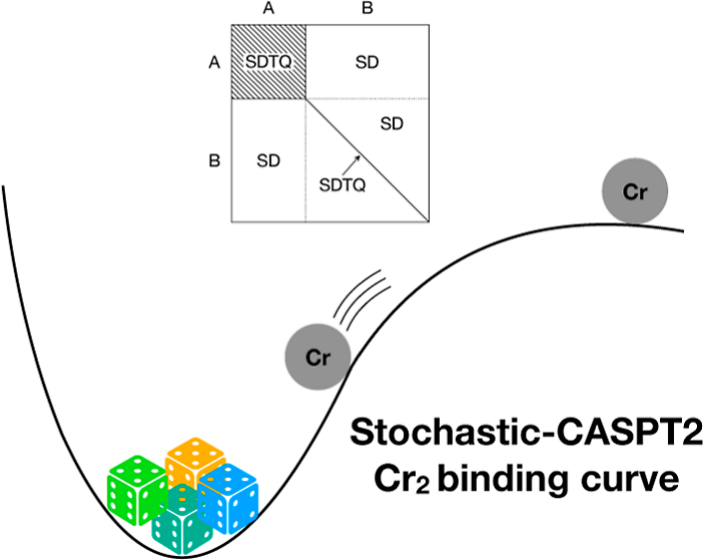

In this work, an internally contracted stochastic complete active space second-order perturbation theory, stochastic−CASPT2, is reported. The method relies on stochastically sampled reduced density matrices (RDMs) up to rank four and contractions thereof with the generalized Fock matrix. A new protocol for calculating higher-order RDMs in full configuration interaction quantum Monte Carlo (FCIQMC) has been designed based on (1) restricting sampling of the corresponding excitations to a deterministic subspace, (2) averaging the RDMs from independent dynamics and (3) projecting them onto the closest positive semi-definite matrix. Our protocol avoids previously encountered numerical conditioning problems in the orthogonalization of the perturber overlap matrix stemming from numerical noise. The chromium dimer CASSCF(12,12)/CASPT2 binding curve is computed as a proof of concept.

## 1 Introduction

In spite of significant progress in the development of full configuration interaction (FCI) solvers, such as the density matrix renormalization group (DMRG)^1−4^ or FCI quantum Monte Carlo (FCIQMC),^5−8^ resource-efficient description of dynamic electron correlation at scale remains an active area of research. The efficacy of capturing static correlation is mainly bound by the flexibility of the many-body basis and a robust method to relax the molecular orbitals. For example, within the stochastic-CASSCF framework,^9^ FCIQMC has been utilized as a CI eigensolver in connection with the super-CI method for orbital relaxation.^10−13^ More recently, stochastic-GASSCF^14^ in the Slater determinant basis and a spin-adapted stochastic–CASSCF^15^ have been developed, complementing existing tools to treat static correlation. Dynamic correlation effects generally require large one-electron bases to converge. One method to recover dynamic correlation is brute-force expansion of the active space. For example, stochastic-MRCISD-like^14^ calculations were successfully performed on a CAS(32,34) reference wave function, correlating 96 electrons in 159 orbitals. With larger basis sets, this strategy becomes computationally inefficient and slow converging with respect to the dynamic correlation. The size of the virtual space also limits the naive application of the uncontracted perturbation theory that uses a perturber space whose size is directly proportional to the length of the reference expansion. The effective Hamiltonian formalism based on Löwdin’s partitioning technique^16^ allows to describe the influence of a perturbation on a model space without changing the dimension of the zeroth-order problem, transferring the computational burden on the calculation of the transfer matrix.^17,18^ Through the model space Quantum Monte Carlo method, this cost can be greatly reduced.^19−21^ Other approaches such as MC-PDFT^22,23^ or transcorrelation^24−27^ promise to alleviate the dependence on the orbital expansion by describing dynamic correlation based on the on-top pair density or Jastrow factors, respectively.

In the realm of pure orbital space methods, internally contracted complete/restricted/generalized active space second-order perturbation theories (XASPT2; X = C, R, and G) remain the most commonly used alternatives to that end.^28−41^ Freezing variational degrees of freedom in the second-order energy functional (“internal contraction”)^42^ in combination with the Cholesky decomposition of the two-electron integrals^43,44^ allow CASPT2 calculations to be routinely performed in the 1000 orbital regime.^45−48^ Due to its cost-to-performance ratio, CASPT2 has seen many new contributions in recent times, ranging from novel intruder state regularization techniques,^49^ to modifications of the zeroth-order Hamiltonian as an alternative to the IPEA shift (IP = ionization potential; EA = electron affinity),^50^ quasi-degenerate variants,^51−55^ and analytic gradients,^54,56^ just to name a few. Solving the CASPT2 equations requires higher-body density matrices conventionally computed from the direct-CI procedure,^57^ limiting the method to active spaces containing 18 electrons in 18 orbitals. Apart from the choice of the zeroth-order Hamiltonian, the accuracy of CASPT2 depends on the reference wave function, since in the state-specific internal contraction formalism the zeroth-order variational degrees cannot relax under the influence of the perturbation.^58,59^ Quantitative accuracy may therefore not be reachable with small active spaces, as was demonstrated for an iron-porphyrin model complex,^60^ as well as [Mn_3_^(IV)^O_4_]^4+^ and [Co_3_^(II)^Er^(III)^(OR)_4_] transition-metal clusters.^23,61^ In light of these limitations, CASPT2 was interfaced to more scalable RAS and GAS reference wave functions.^32,33,62−64^

More recently, CASPT2 has been combined with large CI expansions based on the DMRG.^65,66^ Computation of higher-order density matrices within DMRG is nevertheless costly, hence reduced scaling methods such as the cumulant approximation were explored.^67,68^ Recent applications of DMRG-CASPT2 up to 35 orbitals with and without cumulant approximations were reported in the context of iron-oxo porphyrins and corroles.^69−71^ Hybrid contraction algorithms which leverage the efficiency of existing CI solvers to tackle the dimensionality of the uncontracted perturber space are available as well but only for the Hamiltonians of Dyall and Fink.^72,73^ In a previous attempt to combine CASPT2 with FCIQMC,^74^ Anderson et al. found that uniform stochastic sampling of higher-order excitations introduces large variances into the estimates. When nonlinear operations such as Löwdin orthogonalization are performed on these reduced density matrices (RDMs), sampling errors propagate non-linearly into the PT2 energies.

Here, we present a proof-of-principle stochastic-CASPT2 in the pseudo-canonical orbital basis and demonstrate a potential workflow that circumvents the limitations reported in ref (74) using the example of the CAS(12,12) binding curve. To that end, we rely on a new interface between OpenMolcas(75,76) and the FCIQMC implementation M7.^77^ Our approach is based on three components: (1) confining RDM contributions from determinantal connections of rank three and four to the semi-stochastic space, (2) averaging the RDMs from independent FCIQMC dynamics, and (3) projecting the eigenvalues of the averaged RDMs onto a positive semi-definite set. This paper deals primarily with the identification and solution of problems which prevented a stochastic-CASPT2 in the previous attempt. Work to apply the protocol developed here to systems of practical interest is ongoing and will be presented in a forthcoming publication.

## 2 Theory

The first choice in perturbation theory is related to the partitioning of the original problem as . Since the zeroth-order problem has to be solved exactly, the equations defining  should not be too complicated. CASPT2 was designed as a multiconfigurational generalization of second-order Møller–Plesset perturbation theory and simplifies in the limit of a single configuration to this formalism. Accordingly, Roos and Andersson chose the mono-electronic, generalized Fock operator as the zeroth-order Hamiltonian^29^
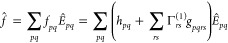
1where *h*_*pq*_ and *g*_*pqrs*_ are the one-electron and anti-symmetrized two-electron integrals,  is the spin-free single-excitation operator, and Γ^(1)^ is the one-body reduced density matrix (1RDM). Due to the CASSCF wave function not being an eigenfunction of the generalized Fock operator, it becomes necessary to project this operator onto the space spanned by the reference, , and its orthogonal complement, (51)
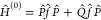
2This usage of projection operators voids the size extensivity of the formalism.^51^ To retrieve a variational estimate of the second-order correction to the energy E_var._^(2)^, the corresponding Hylleraas functional of the first-order wave function, |ψ^(1)^⟩, has to be minimized^78^
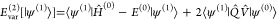
3Both the zeroth-order wave function, |ψ^(0)^⟩, and the corresponding eigenvalue of the zeroth-order operator, *E*^(0)^, have to be known beforehand. This functional is convex in |ψ^(1)^⟩, i.e., the minimum is unique. At stationarity, the gradient vanishes, recovering the expression from Rayleigh–Schrödinger perturbation theory
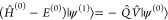
4To keep the cost of the PT treatment minimal, the dimension of the space interacting with the reference wave function, the first order interacting space (FOIS), should be significantly smaller than the FCI. While for a single configuration, the FOIS can be specified unambiguously as single and double excitations from the reference wave function, applying the same approach to each configuration of the active space (“uncontracted PT2”) implies a direct proportionality of the FOIS to the size of |ψ^(0)^⟩, rendering these calculations prohibitive with direct-CI algorithms. Instead, for multi-configurational wave functions, one can show that^79^
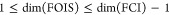
5(Partial) internal contraction^42,80^ is a widespread approach to mitigate the exponential scaling of the uncontracted approach by applying classes of double-excitation operators to the MCSCF wave function as a whole, i.e., freezing the variational degrees of |ψ^(0)^⟩ under the influence of the perturbation. Provided that the perturbation would not cause a significant adjustment of the |ψ^(0)^⟩ amplitudes, reducing the dimensionality of the FOIS does not affect the energy correction substantially; however, if the zeroth-order description is flawed, this approximation breaks down. Practical examples include the *V*-state of ethene or many instances of transition-metal clusters.^23,58,60,61,81^

If the number of holes in the inactive orbitals is denoted with subscripts, the nine possible perturber classes can be compactly denoted by the number of excitations into the external/virtual space.^63^ To distinguish the orbital spaces, we use *i*, *j* for inactive, *t*, *u*, *v*, *x*, *y*, *z* for active, and *a*, *b* for virtual orbitals 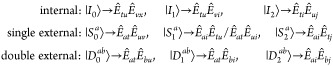
The active–active |*I*_0_⟩ excitation class is only relevant for RAS and GAS expansions, which do not contain all active–active excitations of the corresponding CAS.^10,32,33^ Application of double-excitation operators on the wave function as a whole yields a variational space consisting of linearly dependent states, necessitating orthogonalization of the overlap matrix (“metric”). The number of retained orthogonal states can be controlled through an eigenvalue threshold that is chosen to be on the order of 1 × 10^–8^ in OpenMolcas. Depending on the perturber class, the FOIS metric is defined by the RDMs of rank (4 – *n*) for |*I*_*n*_⟩, (3 – *n*) for |*S*_*n*_^*a*^⟩ and (2 – *n*) for |*D*_*n*_^*ab*^⟩ perturbers.^63^ Expressions for the pertinent matrix elements can be found in the appendix of ref (51). We give one example for the perturber class |*S*_0_^*a*^⟩ (⟨ψ^(0)^|ψ^(0)^⟩ → ⟨⟩)
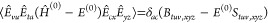
6where **B** and **S** are the contractions of the Fock matrix with the 4RDM (F.4RDM) and the 3RDM as the product of single-excitation operators, respectively
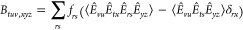
7a
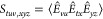
7b

All relevant expressions for a stochastic-CASPT2 can be computed from these two “intermediates”. Given the computational demand of computing the full 4RDM, one can exploit the invariance of CASSCF wave functions under intraspace rotations and eliminate one index in the contraction (7a) by working in the pseudo-canonical basis. Due to the resulting memory advantage, the pseudo-canonical basis is used in the conventional implementation in OpenMolcas; nevertheless, other bases may be better suited for sparse active space solvers such as DMRG^65^ or FCIQMC. In the case of RAS or GAS references, the diagonalization of the entire active block no longer constitutes an invariant rotation, such that classes requiring the 4RDM are either decontracted^63^ or approximated.^32,33^

Two bottlenecks curtail the scalability of the CASPT2 approach.^48^ On the one hand, the construction of the PT2 intermediates is proportional to the number of active orbitals, *n*_act_, as well as the length of the reference wave function in configuration state functions, *n*_CSF_, e.g., constructing the 3RDM is a  process. Accumulation of these tensors within a stochastic framework reduces the dependence on the length of the CI-vector considerably. On the other hand, diagonalizing the overlap matrix of internally contracted functions for the different classes is a  process, where *n* stands for the *n*-body RDM. For perturbers requiring the 3RDM, this procedure becomes expensive with active spaces containing 30–40 orbitals.^71,82,83^ The size of the virtual space impacts the dimension of the perturbation vector ⟨ψ^(1)^|*V*|ψ^(0)^⟩ predominantly via double excitations from the inactive (*i*) to the virtual (*a*) orbitals which scale as . Up to six of these vectors have to be simultaneously held in memory to solve the CASPT2 equations by successive matrix-vector multiplications.^29^ In OpenMolcas, the computational efficiency is increased by representing the two-electron integrals as Cholesky vectors.^47^

### 2.1 IPEA Shift

The initial implementation of the CASPT2 method systematically underestimated the energy correction to closed-shell compared to high-spin states.^84,85^ To rationalize this property,^86^ the generalized Fock operator can be written as a weighted average of IPs and EAs. Whereas for a single-configurational, closed-shell wave function, the eigenvalues of the Fock operator can be linked to IPs and EAs by Koopman’s theorem, this is no longer the case with fractional natural orbital occupation numbers or singly occupied orbitals
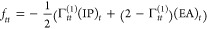
8By adding
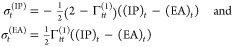
9to the Fock operator in the molecular orbital basis, IPs and EAs are recovered when exciting an electron out of and into a singly occupied orbital, respectively. The separate determination of IPs and EAs is challenging, thus they were combined into an averaged IPEA shift fitted to minimize the mean error of the dissociation energy of 49 small molecules.^86^ A recently explored alternative to the IPEA shift is the inclusion of Koopman matrices into CASPT2.^50^ The eigenvalues of these matrices correspond to the variational estimates of the IPs and EAs of a multiconfigurational wave function.

It is relevant to highlight that the IPEA shift introduces non-invariance under rotations among degenerate active orbitals. Assuming that the PT2 equations are solved in pseudocanonical orbitals, rotations among degenerate orbitals represent the only degrees of freedom. Implemented as a diagonal shift to **B**
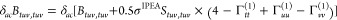
10the IPEA shift introduces non-invariance under degenerate rotations, because the transformation matrices to the orthogonal perturber basis obtained from the diagonalization of *S*_*atuv*,*cxyz*_ = δ_*ac*_*S*_*tuv*,*xyz*_ cannot transform the sums of 1RDMs in the molecular orbital basis. With the standard IPEA of 0.25 *E*_h_, this lack of invariance is often small (≈1 × 10^–4^*E*_h_), but considering recent suggestions in the literature to abandon the notion of an universal shift,^87^ the errors introduced by larger IPEA values could reach the order of magnitude that CASPT2 is trying to resolve. A simple numerical example is the *N*_2_^+2^ cation with a CAS(2,2) consisting of the valence Π_*u*_ orbitals. We generated two sets of active pseudocanonical orbitals by diagonalization of the active-active block of the Fock matrix in the pseudonatural and localized orbital bases. With an IPEA of 0.25 *E*_h_, CASPT2 yields energies of −107.643 946 *E*_h_ and −107.643 834 *E*_h_ (Δ*E* = 1 × 10^–4^*E*_h_), whereas increasing IPEA to 0.75 *E*_h_ results in −107.624 245 *E*_h_ and −107.623 963 *E*_h_ (Δ*E* = 3 × 10^–4^*E*_h_), respectively. Another study which also describes the above-mentioned property of the IPEA shift in the context of CASPT2 analytical gradients.^56^

## 3 Implementation

### 3.1 Sampling of CASPT2 Intermediates

When performing FCIQMC in the basis of Slater determinants with real-valued orbitals, the *n*-RDM, Γ^(*n*)^, is given by the expectation value
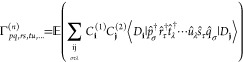
11where the Greek subscripts denote spin and boldface **i**, **j** determinant |*D*⟩ indices. Since the instantaneous walker populations on two determinants have non-vanishing covariance, an unbiased expectation value  has to be computed from two independent replicas, indicated by the number in brackets.^88,89^ Note that in OpenMolcas, the 3RDM elements derived from eq 10 would be indexed as g3(p,q,r,s,t,u), whereas in M7 the creation indices are kept in string order and the annihilation indices are flipped, i.e., g3(p,r,t,q,s,u). RDMs in product-of-single-excitation form (eq 7b) are formed as needed in OpenMolcas through sums with lower-rank RDMs, for example
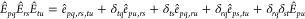
12where  are normal-ordered two-body excitation operators.

Contributions to the off-diagonal RDM elements can be generated (1) during the spawning process, (2) through walkers that themselves do not confer any weight to a determinant (“ghost-walkers”), or (3) by deterministic enumeration within the semi-stochastic space. In the following, we outline each approach.

#### 3.1.1 Spawning

Single excitations, *p* ← *q*, preserving the total spin and point group symmetry, are drawn uniformly in the spawning process. Single excitations from the Hartree–Fock determinant cannot be captured by the spawning process; however, considering the small number of singles from the reference, these contributions are added explicitly by virtue of Brillouin’s theorem. Double connections, *p* ← *q*, *r* ← *s*, are commonly generated with the non-uniform pre-computed heat bath algorithm.^8,90,102^ Non-uniform sampling accrues the benefit of biasing random moves toward high-weighted neighbor determinants that yield large contributions to the RDMs.

#### 3.1.2 Ghost-Walkers

Generating triple connections, *p* ← *q*, *r* ← *s*, and *t* ← *u*, requires special care, as nonuniform excitation generation of triples is prevented by the Hamiltonian not coupling triply-excited determinants. The previous stochastic-MRPT2 implementation^74^ resorted to “ghost”-walkers to uniformly sample these triple- and higher-order excitations without modifying walker weights. Unlike the double connections generated nonuniformly from spawning, drawing unordered pairs of triples or quadruples from the set of determinants defining the CAS space yields small sampling probabilities per RDM element and causes the final estimates to carry large variances. In the inversion of the CASPT2 overlap matrix, this noise propagates non-linearly which made it necessary to modify the FOIS threshold to ensure numerical stability.^74^ For this reason, the concept of ghost-walkers has not been pursued further in the present work.

#### 3.1.3 Deterministic Enumeration in Semi-stochastic Space

**Figure 1:**
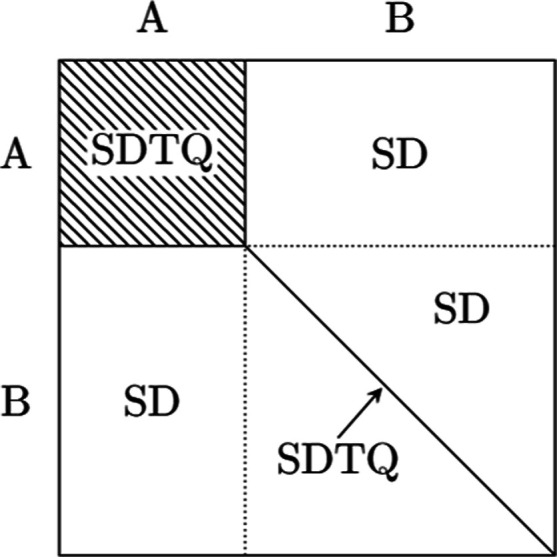
The semi-stochastic subspace partitions the CI space into a principal space (A) containing high-weighted determinants and a residual space (B). Triple and quadruple contributions to higher-order RDMs are only accumulated in the (AA) block, as well as on the diagonal of the (BB) block corresponding to sums of products of instantaneous occupations.

As a compromise between the extremes of exploring the entire triple and quadruple excitation manifold through ghost-walkers (high variance) or omitting them entirely (large truncation error), here we propose sampling a high-weighted subset with reduced variance; see Figure 1.

The semi-stochastic space,^6^ composed of the most important determinants as measured by the instantaneous walker population at initialization, lends itself naturally to this end. Additionally, the sparse map of determinantal connections, already constructed to apply the exact Hamiltonian on this subspace, can be repurposed to enumerate the required RDM contributions. Building upon the results of the first stochastic-CASPT2 study, we suspect that truncating small off-diagonal 3RDM values outside the semi-stochastic space is numerically better conditioned in Löwdin orthogonalization than coarse sampling of all contributions. The error introduced by this approximation depends on the sparsity of the wave function, i.e., it is larger for strongly multi-reference systems than for single-reference ones.

These off-diagonal processes connecting two different determinants do not account for diagonal contributions of the form Γ_*pp*_^(1)^ or Γ_*pq, pq*_^(2)^. Instead, those terms are accumulated by iterating over all instantaneously occupied determinants. Performing this process every iteration would be inefficient; therefore, it is only executed when a walker’s average RDM contributions are computed.^88,89^

All determinantal connections of excitation level *n* yield contributions to the (≥*n*)RDMs; for example, a determinant pair |*D*_**i**_⟩, |*D*_**j**_⟩, such that |*D*_**i**_⟩ = p_σ_^(†)^*q*_σ_|*D*_**j**_⟩, contributes to Γ_*pq*_^(1)^, Γ_*pq,rr*_^(2)^, and Γ_*pq,rr,ss*_^(3)^. The corresponding FCIQMC step is called “promotion”^74^ and constitutes the dominant cost of higher-order RDM estimation, requiring a combinatorially scaling loop over ordered tuples of indices. The most expensive instance of promotion is that of the diagonals of the 4RDM, Γ_*pp,qq,rr,ss*_^(4)^, which necessitates a *n*_elec_ choose four loop every time a walker dies. Partial tracing over the orbital indices due to these loops yields a lower-rank RDM after appropriate normalization, for instance
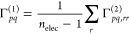
13Contractions with the diagonal Fock matrix can be performed on-the-fly, and we use the same implementation as detailed in the first stochastic-CASPT2 implementation,^74^ such that the 4RDM never has to be stored. Spin tracing and normalization of all tensors are performed at the end of the calculation.^77^

### 3.2 Avoiding Negative Eigenvalues in the 3RDM

The RDMs of all orders fulfill a hierarchy of N-representability conditions to ensure that they correspond to a N-electron wave function.^91,92^ For the 1RDM, these amount to positive semi-definiteness (PSD) and natural occupation numbers less than or equal to two. For all higher-rank RDMs, the conditions may be constructed by considering the convexity of the set of N-electron RDMs.^91^ Important for our purposes is that RDMs of all orders retain the PSD property as a necessary but not sufficient N-representability condition. As we show in the next section, PSD violations of the 3RDM correlate with numerical instabilities of the stochastic-PT2 method; hence, a method to ensure this property is important.

In FCIQMC, the exact wave function only is sampled on average in the infinite sampling and walker limit. In the context of RDM accumulation, this property implies that each individual snapshot of the wave function is not guaranteed to be physically meaningful, and negative eigenvalues can occur in the RDMs. Enforcing N-representability conditions at the sampling stage is difficult, since information on the eigenvalue distributions of the RDMs would have to be incorporated into the walker dynamics.^88^ With a proper parametrization, the averaged stochastic estimates are usually close enough to the exact solution and as a consequence of linearity, expectation values are rather insensitive to errors. Following this argument, stochastic errors in the F.4RDM, which occurs linearly in the PT2 equations, have only a small impact on the retrieved energy. Conversely, removing linear dependencies in the perturber space requires diagonalizing and inverting the FOIS metric, both non-linear operations sensitive to errors in the original tensor.

Numerically, we have observed an increase in negative eigenvalues proportional to the rank of the sampled RDM. In the present work, we address this problem by finding the closest PSD matrix in the Frobenius norm, which preserves the RDM trace
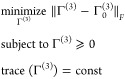
14This task is a convex optimization problem, solvable at the asymptotic cost of diagonalizing the 3RDM . Considering that a similar operation is required to perform Löwdin orthogonalization in CASPT2, this overhead does not impair the scalability of our implementation. In the Supporting Information, we provide one algorithm reported in the literature that has been used here to find PSD 3RDMs.^93^ As shown in the following, this simple PSD purification consistently improves upon the nonpurified 3RDMs in terms of CASPT2 energies, although it should not be used to recover from catastrophic failure in sampling.

Averaging RDMs from multiple, statistically uncorrelated calculations is another key strategy to reduce the magnitude of negative eigenvalues. Inspired by the replica trick for sampling RDMs from instantaneous populations, we approximated the expectation value of an averaged wave function by an average of truncated expectation values.

Assuming that the CI coefficients correspond to the components of the exact Hamiltonian eigenvector, the truncation of the excitation level conserves N-representability. For example, RDMs derived from wave functions truncated at any excitation order are invariably PSD.^78^ Exact CI coefficients are available from FCIQMC only by computing time-averaged walker distributions. Histograming all determinants of the Hilbert space voids the memory advantage of the method, which is why expectation values are usually computed from instantaneous occupations. In contrast to the replica method, where in the infinite walker limit the entire Hilbert space is occupied, the truncation error in the subspace approximation can only go to zero if the semi-stochastic space comprises the FCI space. Therefore the truncation error can not be mitigated through RDM averaging; however, the stochastic error that causes positivity violations goes to zero when more RDMs are used in the average. Compared to sampling the CASPT2 intermediates longer, averaging over uncorrelated compositions of the semi-stochastic space converges the stochastic error more rapidly, as will be shown in the next section.

We observed that for different initializations of the random number generator, the severity of PSD violations varied. Since the arithmetic mean with small sample sizes is susceptible to outliers, we conceived an outlier detection scheme instead of averaging all available RDMs. To this end, we found a *z*-score criterion to work well in practice. Let *x* be an array of values and |*x*| the element-wise absolute value, then values are rejected as outliers if
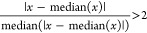
15For the purpose of the analysis in Section 4.3, outliers in the 3RDMs are distinguished by the magnitude of their largest negative eigenvalue.

Despite the lower sensitivity of the F.4RDM to stochastic noise, we encountered instances where, even with the exact 3RDM, discontinuities in the binding curve arose. In light of the high cost associated with sampling F.4RDM, we turned to a combination of averaging and the outlier detection scheme to reduce the noise of the estimates.

**Figure 2:**
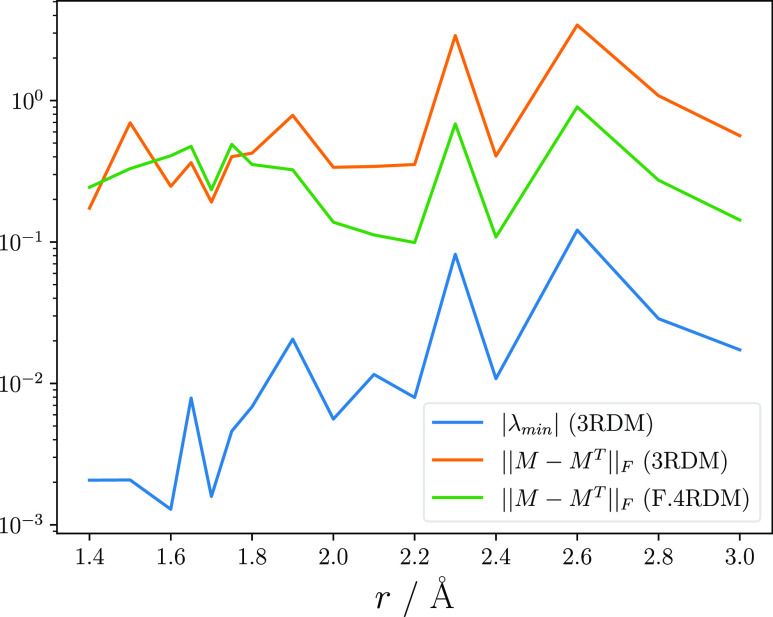
Comparison of the hermiticity error of the averaged F.4RDM and 3RDM, as well as the largest negative eigenvalue of the averaged 3RDM from six independent runs sampled for 20k iterations.

Unlike the 3RDM, we are not aware of analytical bounds on the eigenvalue distributions of the F.4RDM. Additionally, pseudo-random number generator (PRNG) seeds with poorly converged 3RDMs turned out to be likely but not guaranteed outliers in the F.4RDM, rendering elimination based solely on the eigenvalue criterion ineffective. Numerically, we identified the hermiticity error of the 3RDM as an alternative indicator having strong positive correlation with the largest negative eigenvalue (*p* = 0.98), see Figure 2. The positive correlation between the hermiticity error of the 3RDM and the F.4RDM (*p* = 0.81) suggests that this indicator can be generalized to identify outliers in the F.4RDM.

Hermiticity violations of FCIQMC RDMs result from an algorithmic simplification not to add contributions symmetrically and are usually accounted for in post-processing. It is important to note that the lack of hermiticity is only utilized in the outlier detection scheme, and all tensors are symmetrized before usage in CASPT2.

## 4 Application

The computation of the chromium dimer binding curve has historically served as a benchmark for electronic structure methods.^18,83,94−96^ This example is particularly interesting, because the sparsity of the CI-solution varies smoothly along the binding curve and provides an opportunity to assess the properties of the semi-stochastic subspace triples and quadruples in different correlation regimes.

### 4.1 Computational Details

For the *X*(^1^Σ_*g*_^+^) state of the chromium dimer, the minimal active space is the CAS(12,12) consisting of the 4s and 3d orbitals on each atom. Around the equilibrium bond distance, the system is loosely described by the closed-shell Hartree-Fock solution. For example, at 1.65 Å, the Hartree-Fock determinant carries 51% weight. As the bond is stretched, the sparsity of the CI vector decreases smoothly to the limit of no unique reference configuration in a delocalized orbital basis. At 3.0 Å, the Hartree-Fock determinant has approximately 0.6% weight. For all geometries, we used CASSCF(12,12) pseudo-canonical orbitals, the ANO–RCC basis set^97^ in triple-ζ quality with contractions Cr(21s15p10d6f4g)/[6s5p3d2f1g] and the *C*_1_ point group. Comparative DMRG^83^ and RASPT2^95^ studies found a significant dependence of the CASPT2 potential energy curve on the parametrization of the zeroth-order Hamiltonian and choice of active space. Here, the IPEA and imaginary shifts were chosen as 0.45 *E*_h_ and 0.2 *E*_h_, respectively, which are known to yield an attractive, intruder-state-free binding potential with the CAS(12,12) reference.^18^ RDM accumulation in FCIQMC is independent of these corrections, and arbitrary values could have been used, but this choice simplifies the distinction of intruder states due to our method from genuine intruders occurring with numerically exact RDMs. We utilized the CAS(12,12) to discuss the numerical challenges introduced by stochastically sampled RDMs and show how our novel procedure circumvents these limitations. At the same time, reference energies and RDMs for the minimal CAS(12,12) are available and easy to analyze. Unless noted otherwise, the walker population was grown to 1 M walkers (*N*_*w*_), a small number for a FCIQMC dynamic, and left to equilibrate for 5k iterations before the initialization of the latter. The size of the semi-stochastic space plays a leading role in the computational cost of the procedure, hence sizes ranging from 5k to 12.5k determinants were probed. Sampling of the 3RDM and F.4RDM commenced 7.5k iterations after the construction of the semi-stochastic space. The initiator adaptation with a threshold of three was used in all calculations.^7,8,98^ This parametrization proved sufficient to converge the variational energy estimate to sub-mHa accuracy.

### 4.2 Binding Curve

**Figure 3:**
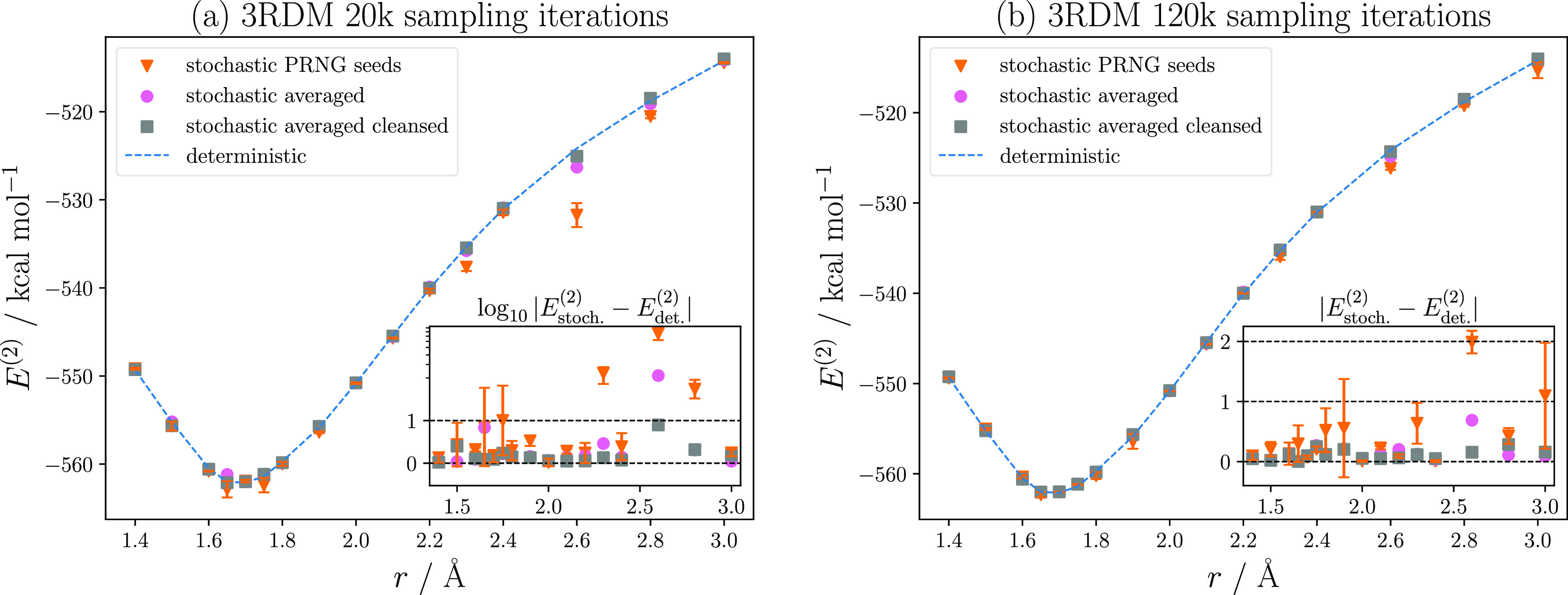
Comparison between the binding curves obtained with 20k and 120k 3RDM sampling iterations. See the text for details.

In Figure 3, we report the Cr_2_ binding curves as obtained with different sampling durations and post-processing methods.

Shown in Figure 3a are the binding potentials obtained from sampling the 3RDM and F.4RDM for 20k iterations. To gauge the error associated with an individual run, the standard error was computed from six uncorrelated calculations (orange triangles). The binding curve derived from these runs was not smooth and the associated standard error (orange bars) for different initializations (“seeds”) of the PRNG considerable. As shown in the inset, the magnitude of the standard error is not a simple function of the bond length and also for the 1.65 Å and 1.75 Å geometries larger than 1 kcal mol^–1^ (“chemical accuracy”). Upon averaging the CASPT2 intermediates of these calculations (pink circles), the proper shape of the potential was restored; only at 2.6 Å was the deviation 2 kcal mol^–1^. That adjustments of the FOIS linear dependency threshold were not necessary to this end is a remarkable improvement over the previous attempt.^74^

In the Supporting Information we analyze the difficulties for the FCIQMC algorithm that are associated with the 2.6 Å geometry. The PSD purification (gray squares) further reduced the two remaining “kinks” at 1.65 and 2.6 Å, reaching 1 kcal mol^–1^ across the entire curve. Notably, for points between 1.8 and 2.2 Å, which were already well described with seed-specific RDMs, neither averaging nor PSD purification had a negative impact on the results.

That the resolution of the 3RDM determines the accuracy of stochastic-CASPT2 was confirmed by increasing the 3RDM sampling duration from 20k to 120k and averaging twelve individual 3RDMs while leaving the parametrization of the F.4RDM constant. The corresponding binding curves are shown in Figure 3b using the same marker convention. For different PRNG seeds, the overall curve follows the reference better, but as the inset shows the standard errors for 1.9 Å and 3.0 Å remain larger than 1 kcal mol^–1^. Chemical accuracy can only be obtained with a combination of RDM averaging and PSD purification. Importantly, sampling individual seeds longer does not ensure a smaller standard error, due to the non-linear error propagation in the CASPT2 equations. Consider for instance the point at 3.0 Å, where the standard error is an order of magnitude larger after 120k iterations than for 20k iterations. Averaging RDMs lessens the impact of sampling duration, yielding benchmark results in both cases. Comparing the results from averaging six 3RDMs sampled for 20k iterations with the ones from twelve averaged RDMs sampled for 120k iterations suggests that increasing the number of averaged seeds beyond six or increasing the sampling iterations of the F.4RDM provides only diminishing return.

### 4.3 Negative Eigenvalues as Proxies for Sampling Quality

**Figure 4:**
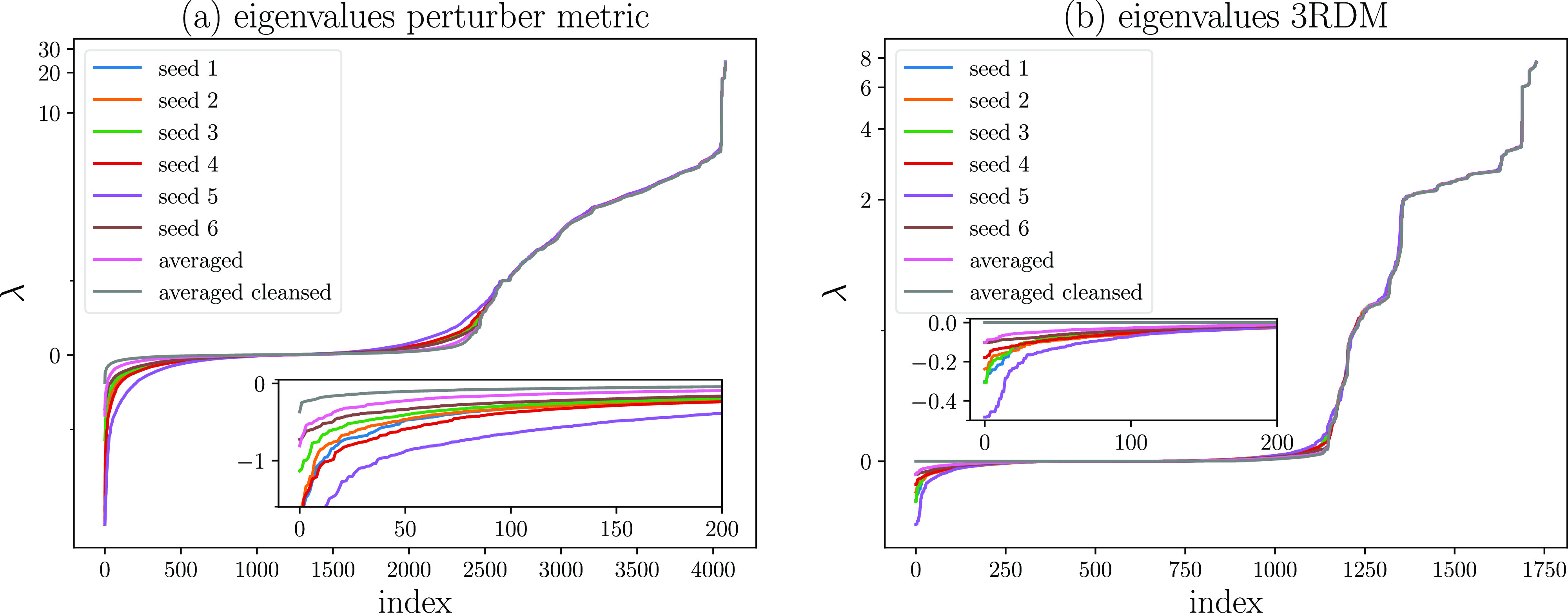
Eigenvalue distribution of the (a) FOIS overlap matrix across all perturber classes and (b) underlying 3RDMs sampled for 20k iterations at the 2.6 Å geometry.

In the following, we establish a correspondence between the standard error obtained for different PRNG seeds and the properties of the PT2 metric. Shown in Figure 4 are the eigenvalues of the FOIS overlap matrices over all perturber classes sorted by magnitude (Figure 4a) and of the 3RDM as retrieved from the FCIQMC (Figure 4b).

The most prominent feature of the curves is the seed–dependent distribution of negative eigenvalues, which stem almost exclusively from classes requiring the 3RDM. Upon averaging the individual 3RDMs (pink lines), their magnitude is reduced compared to those of all seed-specific distributions. Further improvements can be realized by enforcing the PSD property of the 3RDM (gray lines). Note that small negative eigenvalues remain in the FOIS overlap spectrum, since the purification only enforces the PSD property of the 3RDM. Strict positivity of the perturber metric can only be achieved if the 3RDM is N-representable, see Section 3.2.

### 4.4 Impact of FCIQMC Parametrization on CASPT2 Energy

**Figure 5:**
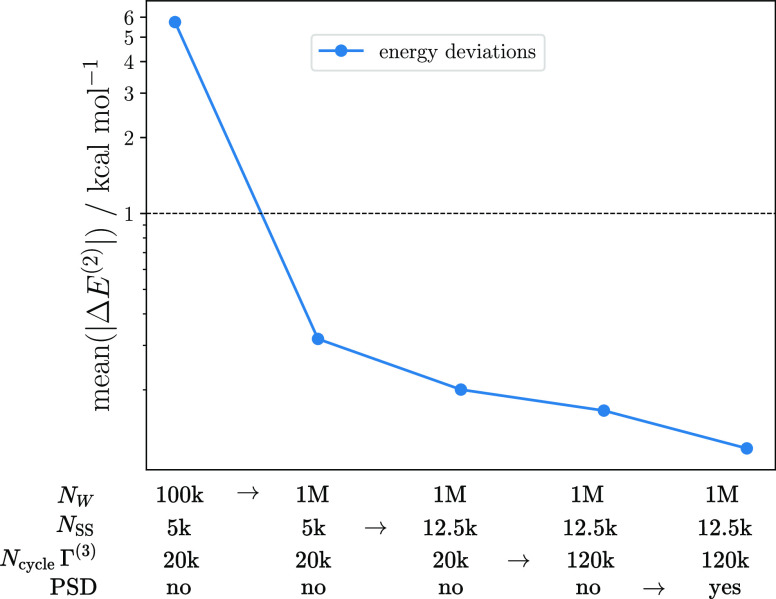
Mean of the absolute errors across the binding curve of the stochastic-CASPT2 energies.

As shown in Figure 5, we benchmarked the protocol in terms of total number of walkers, size of the deterministic subspace (*N*_SS_), number of 3RDM sampling iterations, and usage of the PSD purification. The number of averaged seeds and F.4RDM sampling iterations are constant at six and 20k, respectively, because we found these parameters to be already well converged.

The plot illustrates that the resolution of the wave function, as facilitated by a sufficient number of walkers, is the most important criterion to achieve chemical accuracy with a moderate number of sampling iterations. While for this system, 1 M walkers were sufficient to converge the CI problem, for larger systems up to 4 billion walkers were reported^99^ and could be used to perform larger stochastic-CASPT2 calculations. Increasing the size of the semi-stochastic space also has a positive, but not as important effect on the average deviation, suggesting that the highest-weighted contributions of the CAS(12,12) are already well described with a small subspace. This result is particularly encouraging since the number of RDM-contributing determinantal connections scales steeply with the size of the subspace. To retain the same accuracy upon increase of the active space, the size of the deterministic space must be adjusted such that the highest-weighted determinants are retained in the subspace. The exact scaling law depends on the structure of the wave function, i.e., the exponent will be higher for strongly multireference than for single-reference wave functions. For a given sampling granularity, the stochastic error can always be reduced by increasing the number of sampling iterations; however, in regimes where more than 20k 3RDM iterations are not computationally feasible, the PSD purification may be the more economic route to achieve higher accuracy.

The chromium dimer dissociation curve is known to be susceptible to intruder states when cumulant approximations on the RDMs are imposed.^67^ We encountered vanishing reference weights only when low walker populations were combined with small semi-stochastic spaces, for instance 100k walkers with a semi-stochastic space of 5k determinants. In these cases, already the CAS-CI energy was highly oscillatory and RDMs from such ill-behaved dynamics were not converged. For larger active spaces, intruders in stochastic-CASPT2 despite converged CAS-CI dynamics may occur, but examples will require more experience with the method in realistic regimes.

**Figure 6:**
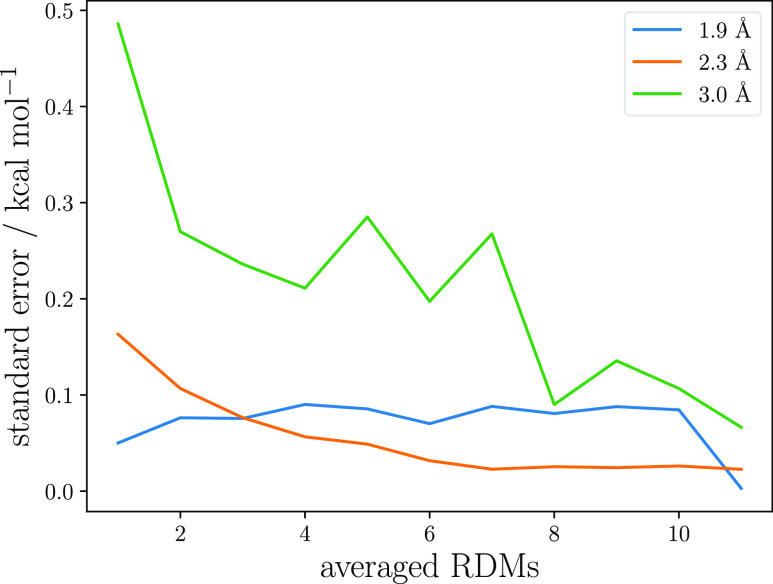
Estimates of the standard error obtained by resampling 3RDMs from 12 seeds sampled for 120k iterations for the 1.9, 2.3, and 3.0 Å geometries.

An estimate of the error on the CASPT2 energy caused by averaging can be obtained through the statistical technique of resampling.^100^ In one variation of this method, members of an existing population are chosen randomly with replacement, and an average is computed. Multiple repetitions generate a set of averages that can then be used to obtain an estimate of the standard error. Here, we consider the 1.9, 2.3, and 3.0 Å geometries that have the largest standard error for different PRNG seeds in Figure 3b. Leaving the F.4RDM constant, twelve sets of 3RDM averages consisting of a variable number of 3RDMs were formed and the standard error from the corresponding CASPT2 energies was computed. The results are shown in Figure 6. Note that the standard errors for the twelve different PRNG seeds are not the same as the one computed with six seeds in Figure 3b.

Although not strictly monotonic, the smallest error is invariably achieved for the largest number of 3RDMs included in the average. In agreement with our previous conclusion, the decay is rapid and four to six RDMs already prove sufficient to reduce fluctuations in the CASPT2 energy to 0.2 kcal mol^–1^. Beyond this point, averaging more 3RDMs provides only diminishing returns.

## 5 Conclusions

We have presented a new method to compute the 3RDM and F.4RDM as required for CASPT2 in FCIQMC by combining single and double contributions from the entire space with triple and quadruple excitations only accumulated in the semi-stochastic space. Stochastic sampling was shown to cause negative eigenvalues in the perturber metric, the magnitudes of which depend strongly on the PRNG seed of the calculation. By averaging multiple statistically uncorrelated estimates and projecting the resulting 3RDM eigenvalues onto a convex set, their magnitude can be reduced significantly, as shown in Figure 4. Moreover, averaging RDMs reduces the variance of the obtained energy estimate, see Figure 6, more effectively than increasing the sampling duration on a single FCIQMC run. Combined with a proper parametrization of the FCIQMC dynamic, the new workflow reproduces the chromium dimer CASSCF(12,12)/CASPT2 binding curve to sub-kcal mol^–1^ accuracy. While modest in absolute scale, these results are a major step forward with respect to earlier attempts and a promising proof of concept for more sizable applications.

Despite the fact that stochastically sampled higher-order RDMs within the graphical unitary group approach are currently unavailable, spin-pure stochastic-CASPT2 may still be realized by combining the presented algorithm with a first-order spin purification technique.^101^

Our results on the dependence of the PT2 energy with respect to the number of walkers show that for larger systems pseudocanonical orbitals will require a high number of walkers to achieve stable FCIQMC dynamics, as a byproduct increasing the cost of sampling the CASPT2 intermediates. Qualitatively similar conclusions were drawn in previous DMRG–CASPT2 implementations.^65,68^ Working in a one-particle basis more conducive to FCIQMC introduces the complication of having to sample the contraction of the full 4RDM with a non-diagonal Fock matrix. Approaches to overcome the computational bottleneck of looping over eight-index quadruple excitations are currently under development.

## Supporting Information Available

CASSCF(12,12) natural orbitals for all geometries, Python3 scripts to average and purify the 3RDM and F.4RDM, CASSCF and stochastic-CASPT2 energies pertaining to the binding curve, as well as F.4RDM hermiticity errors and 3RDM largest negative eigenvalues formatted as numpy arrays, and two input examples for M7 and OpenMolcas (ZIP)
Description of the PSD purification algorithm and details on the annihilation plateau across the binding curve (PDF)

## Notes

Published as part of *The Journal of Physical Chemistry A*virtual special issue “Roland Lindh Festschrift”.
