# Supplementary material for: Toward a Stochastic Complete Active Space Second-Order Perturbation Theory
Source: J Phys Chem A. 2023 Dec 28;128(1):281–91. doi: 10.1021/acs.jpca.3c05109 (PMC10788896; doi:10.1021/acs.jpca.3c05109)
Supplement: Supplementary file 2 — jp3c05109_si_002.pdf [file jp3c05109_si_002.pdf]

Supplementary Information: Towards a Stochastic Complete Active Space Second Order Perturbation Theory

Arta A. Safari, Robert J. Anderson, and Giovanni Li Manni*

Max-Planck-Institute for Solid State Research, 70569 Stuttgart, Germany

E-mail: g.limanni@fkf.mpg.de

S1 PSD Purification Algorithm

Algorithm 1 PSD purification algorithm adopted from Harada.[1] The procedure can be interpreted as gradient ascent on the dual function of the original problem with step size $\frac{1}{\text{dim}}$.

$\Gamma_0^{(3)} = \sum_i^{\text{dim}} |i\rangle \, \lambda_i \, \langle i|$
$\vec{e} \leftarrow (1, 1, ..., 1)^T$
repeat
$\quad \delta \leftarrow \frac{1}{\text{dim}} \left(\text{Tr}\left(\Gamma_0^{(3)}\right) - \sum_i^{\text{dim}} \max(\lambda_i, 0) \right) \vec{e}$
$\quad \lambda_i \leftarrow \lambda_i + \delta$
until $\|\delta\| \leq 1 \times 10^{-10}$
return $\tilde{\Gamma}^{(3)} \leftarrow \sum_i^{\text{dim}} |i\rangle \max(\lambda_i, 0) \langle i|$

S2 Annihilation Plateau across the Binding Curve

A metric to assess the difficulty of the CI problem for FCIQMC in a given orbital basis is to compute the minimum number of walkers required to stabilize a specified population on the reference determinant. In Figure S1 this "saturation plateau" is shown across the binding curve for 100 N_w with and without the initiator adaptation.

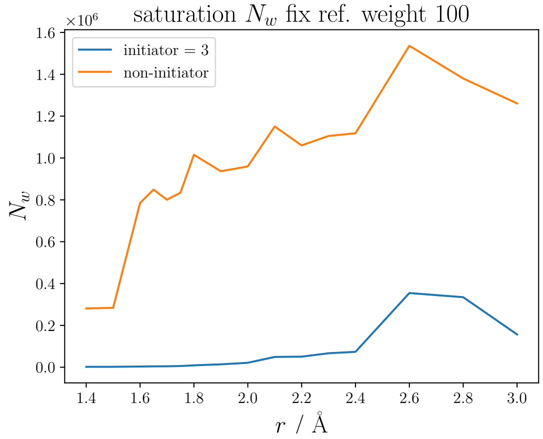

Figure S1: Population in units of millions required to stabilize one hundred walkers on the chosen reference determinant. See text for details.

Both curves obtain their maximum at 2.6 Å which is the geometry with the highest

error relative to the reference CASPT2 energy. Importantly, for the stretched geometries, obtaining stable FCIQMC dynamics with the Hartree–Fock determinant as a reference proved difficult and beyond an inter atomic distance of 2.3 Å an alternative reference ensuring sub–mHa convergence of the CAS–CI energy was chosen. Monotonicity in the saturation plateau for this piece of the binding curve is therefore not to be expected; nevertheless, even in regions where the HF determinant was chosen, the number of required walkers is not strictly monotonic. Notably, with the initiator adaptation monotonic behavior in the Hartree–Fock dominated region can be restored.

References

(1) Harada, K. Positive semidefinite matrix approximation with a trace constraint. *NTT-DATA Math. Syst. Inc., Tokyo, Japan, Rep* **2018**, *6765*.
